# The Intertwined Relationship Between an Overactive Thyroid and an Overactive Mind: A Case Report and Review of Literature

**DOI:** 10.7759/cureus.50748

**Published:** 2023-12-18

**Authors:** Sushma Bilichodu Rangappa, Aditi Sharma, Sreekant Avula, Deepak Chandramohan, Vinod Sharma

**Affiliations:** 1 Psychiatry, Chamarajanagar Institute of Medical Sciences, Mysore, IND; 2 Psychiatry, The Wright Center for Graduate Medical Education, Scranton, USA; 3 Diabetes, Endocrinology, and Metabolism, University of Minnesota, Minneapolis, USA; 4 Internal Medicine and Nephrology, University of Alabama at Birmingham, Birmingham, USA

**Keywords:** graves, hyperthyroid, thyrotoxicosis, hormones, psychosis, mania, thyroid

## Abstract

This case report presents the clinical scenario of a 45-year-old male patient who exhibited acute psychiatric symptoms as the initial manifestation of Graves' disease, a common etiology of hyperthyroidism. The patient presented with severe agitation, persecutory delusions, and auditory hallucinations, raising concerns about his mental health. Detailed diagnostic evaluations revealed thyroid dysfunction characterized by markedly low thyroid-stimulating hormone (TSH) levels, elevated free T4 levels, and increased total T3 levels, indicative of thyrotoxicosis. Elevated thyroid-stimulating immunoglobulin (TSI) levels further confirmed the diagnosis of Graves' disease. The patient received treatment with methimazole and propranolol to manage the hyperthyroidism, leading to the resolution of psychiatric symptoms. This case emphasizes the importance of considering thyroid function in patients presenting with acute psychiatric disturbances. This literature review explores the intricate relationship between hyperthyroidism, a condition characterized by the excessive production of thyroid hormones, and its impact on psychological and cognitive processes. Understanding the connection between an overactive thyroid and an overactive mind is crucial for clinicians and researchers to provide comprehensive care and treatment for affected individuals.

## Introduction

Graves' disease is a well-recognized cause of hyperthyroidism, often associated with a spectrum of physiological and psychological symptoms [1]. However, the occurrence of acute psychiatric manifestations as the primary clinical presentation is relatively infrequent [2]. This case report underscores the significance of recognizing the intricate relationship between thyroid function and psychiatric symptomatology, emphasizing the need for comprehensive diagnostic assessment in patients presenting with acute psychiatric disturbances. Hyperthyroidism, often an overactive thyroid, is a common endocrine disorder characterized by excessive production of thyroid hormones, primarily thyroxine (T4) and triiodothyronine (T3). While the physical symptoms of hyperthyroidism are well documented, such as weight loss, tremors, and tachycardia, its effects on mental health are often overlooked [1-3]. This case report aims to provide an overview of the relationship between hyperthyroidism and the development of psychosis, shedding light on the complex interplay between an overactive thyroid and an overactive mind. The connection between thyroid dysfunction and mental health has been a subject of interest in the medical community for many years. While the association between hypothyroidism and psychiatric symptoms is well-established, there is a growing body of evidence suggesting a strong link between hyperthyroidism, particularly in the form of Graves' disease, and acute psychosis [3,4].

This article was previously presented as an abstract at the ENDO 2023 Annual Scientific Meeting on June 16th, 2023 [5].

## Case presentation

A 45-year-old male with no significant past medical or psychiatric history was brought to the emergency department (ED) by police after he was found to be walking in front of a shopping mall and was severely agitated. On arrival at the ED, he was found to be very restless, irritable, and aggressive. The patient was found to have persecutory delusions and auditory hallucinations. His blood pressure was elevated, and he had tachycardia. He was given haloperidol to calm down. The patient was evaluated with blood work and urine toxicology. The physical examination was unremarkable, and he had no family history of psychiatric illness and substance abuse disorder.

Laboratory findings

The diagnostic workup unveiled strikingly low levels of thyroid-stimulating hormone (TSH; <0.01 mIU/L), elevated free T4 levels of 4 ng/dl (0.7-1.5 ng/dl), and increased total T3 levels of 380 ng/dl (80-200 ng/dl), consistent with thyrotoxicosis. Further investigations revealed elevated levels of thyroid-stimulating immunoglobulin (TSI) of 32 IU/L (</= 0.54 IU/L), substantiating the diagnosis of Graves' disease. The urine drug screen was negative.

Management and treatment

To address the hyperthyroidism, the patient promptly started a treatment regimen consisting of methimazole 10 mg three times a day and propranolol 10 mg three times a day. His psychiatric symptoms got better in three days after starting methimazole. Once the patient was stable, he was further interviewed; he gave a history of palpitations, increased anxiety, and weight loss for two months, but he didn’t seek any medical attention. By the time of discharge, which was 10 days since admission, he had symptomatically improved, and his free T4 and total T3 had trended down significantly. As the patient's thyroid function normalized, the psychiatric symptoms progressively ameliorated, leading to his discharge with a prescription for methimazole 10 mg and propranolol 10 mg three times a day as needed. The patient was scheduled for a follow-up with an endocrinologist in one month.

## Discussion

Epidemiology

It is not uncommon for individuals with Graves' disease and other forms of thyrotoxicosis to display subtle neuropsychiatric abnormalities such as irritability, anxiety, sleep disturbances, confusion, mood changes, and memory deficits [4]. In the United States, thyrotoxicosis affects 1.2% of the population, with 0.5% overt cases and 0.7% subclinical cases [6]. Its highest incidence occurs between ages 20 and 50 [6]. Graves' disease is the most common cause, affecting primarily women aged 30 to 50 (a male-to-female ratio of 5:1) [6]. However, the occurrence of psychosis as the primary presenting feature of thyrotoxicosis is exceptionally rare. The epidemiological data on the psychiatric manifestations of Graves' disease is limited. Von Basedow first reported a case of manic psychosis in a Graves' disease patient [7]. Brownlie et al. found in a Christchurch study that 1% of thyrotoxicosis patients over a 20-year period required psychiatric treatment, predominantly involving antipsychotic medications and thionamides [8]. In a study by Lo et al., four out of 22 patients with organic delusional disorders had psychosis attributed to hyperthyroidism [9]. Consistent findings in the literature emphasize the importance of thyroid testing for patients presenting with psychiatric symptoms in the acute emergency department. It is noteworthy that thyrotoxicosis is now acknowledged as a potential cause of organic psychosis [7-10].

Psychiatric symptoms in hyperthyroidism

Traditionally, psychiatric manifestations were predominantly linked to hypothyroidism. However, emerging research has revealed that hyperthyroidism, characterized by an excess of thyroid hormones, can also lead to a wide range of psychiatric symptoms [3,4]. Patients with thyrotoxicosis, the state of excessive thyroid hormone production, may exhibit symptoms such as anxiety, restlessness, emotional lability, depression, mania, and, in rare cases, acute psychosis, paranoia, and schizophreniform features [1,4]. Patients with Graves’ disease, an autoimmune thyroid disorder characterized by hyperthyroidism, often exhibit subtle neuropsychiatric abnormalities, including irritability, anxiety, sleep disturbances, confusion, mood changes, and memory deficits [2,7]. A report of a case series of 18 patients of newly diagnosed thyrotoxic patients with acute psychosis was published by Brownlie et al. in 2000 [8]. It was a retrospective study of 18 patients who required inpatient psychiatric care between 1973 and 1993 at Christchurch Hospital, New Zealand. In this case series, out of 18 patients, 16 patients were psychiatrically ill when first seen in the thyroid clinic, and the remaining two patients developed psychosis after they were started on antithyroid medication with an associated fall in thyroid hormone levels. Most patients were females (16 patients), and males accounted for two. None of the patients had any past history of psychiatric illness, which was consistent with our patient, who also didn’t have any previous psychiatric illness. Of the 18 patients, the majority were classified as having affective psychosis based on ICD9 criteria (mania and major depression); others had schizophreniform psychosis, paranoid psychosis, and delirium. All 18 patients received thionamide antithyroid medications (carbimazole) for thyrotoxicosis. Once they were euthyroid and their psychiatric illness well controlled, they were offered definitive treatment with I131 radioiodine ablation, but only 16 patients agreed, and they never had any relapse of psychiatric illness. Two patients who refused definitive treatment had episodes of relapse of psychiatric illness.

Hyperthyroidism and psychosis

Hyperthyroidism has been associated with a range of neuropsychiatric symptoms, including anxiety, depression, and cognitive impairment [6,7]. However, the manifestation of psychosis in hyperthyroid individuals is less common but not unheard of [4]. Psychosis in hyperthyroidism is characterized by hallucinations, delusions, and thought disorders [7]. Several case reports and small studies have documented the presence of psychosis in hyperthyroid patients, but the exact mechanisms underlying this relationship remain unclear [8].

Pathophysiology of psychosis in thyrotoxicosis

The precise mechanism that underlies the development of psychosis in Graves’ disease and other forms of hyperthyroidism remains elusive. One hypothesis is that the overstimulation of the central nervous system by excess thyroid hormones may lead to heightened excitability in neural circuits, potentially triggering psychotic symptoms [10]. Another proposed mechanism is the role of autoimmune processes and inflammation in the brain, as hyperthyroidism can be associated with autoimmune thyroiditis, which may affect the central nervous system [11,12].

Nonetheless, it is known that a significant number of thyroid hormone receptors are located in the brain, particularly in the limbic system, which includes regions like the hippocampus and amygdala [11]. These receptors influence various functions, including behavior, mood, and long-term memory [12]. It is postulated that excess thyroid hormones can impact neurotransmitter activities, such as serotonin and dopamine, within the brain's limbic system, potentially contributing to the neuropsychiatric abnormalities observed in thyrotoxicosis [12]. Additionally, thyroid hormones can modulate the beta-adrenergic response to catecholamines within the central nervous system, which may also play a role in the development of psychotic behaviors in thyrotoxic patients [11,12].

It is important to note that hyperthyroxinemia can be a consequence of psychiatric disorders as well. This form of hyperthyroxinemia is characterized by a modest and often temporary elevation of thyroid hormones and TSH within the upper range of normal [13]. This alteration in thyroid function can also occur in non-thyroidal disorders and is commonly referred to as "euthyroid sick syndrome" [13].

Treatment

The management of hyperthyroidism-related psychosis and mania typically involves addressing the underlying thyroid dysfunction through antithyroid medications, radioactive iodine therapy, or surgery [14]. The preferred approach for treating hyperthyroidism, along with the associated psychiatric disorders and mental symptoms, involves a combination of antithyroid drugs and β-adrenoceptor antagonists [11]. Despite successful hyperthyroidism treatment, a notable number of patients exhibit persistent altered mental states, indicating potential involvement of mechanisms beyond hyperthyroidism itself, such as Graves' autoimmune process and ophthalmopathy [15]. In cases where psychiatric disorders persist even after achieving euthyroidism and administering β-adrenoceptor antagonists, specific treatments for psychiatric symptoms, including psychotropic drugs, may be necessary [15]. In cases where psychiatric symptoms are severe, antipsychotic medications may be prescribed to manage psychosis [16]. However, monitoring thyroid hormone levels closely during treatment is essential, as the correction of thyroid dysfunction may ameliorate psychiatric symptoms [15].

Strengths and limitations

This case report and literature review on psychiatric manifestations associated with Graves' disease present notable strengths and limitations. The inclusion of historical context and incorporation of different studies contribute to diverse perspectives on the prevalence and treatment approaches for psychiatric symptoms in thyrotoxicosis patients. However, a limitation arises from the limited epidemiological data, underscoring the need for more extensive research to enhance our understanding of the intricate relationship between Graves' disease and psychiatric manifestations. Further research is crucial to draw more definitive conclusions regarding the prevalence and optimal treatment strategies for psychiatric symptoms associated with Graves' disease.

Future research and implications

This literature review underscores the need for further research to elucidate the complex interplay between excess thyroid hormones and psychiatric disorders. A better understanding of the pathophysiological processes involved in thyrotoxicosis-related psychosis can aid in developing clear treatment guidelines, reducing morbidity, and potentially eliminating the need for long-term psychiatric medications. Identifying somatic causes for psychiatric issues, as demonstrated in this case, is essential to promoting holistic patient care and fostering complete recovery.

## Conclusions

The relationship between hyperthyroidism and psychosis is complex and multifaceted, with underlying mechanisms that remain a subject of ongoing research. Recognizing the psychiatric manifestations of hyperthyroidism is crucial for timely diagnosis and intervention, as untreated thyroid dysfunction can lead to severe psychological distress and functional impairment. This case report and literature review underscore the importance of interdisciplinary collaboration between endocrinologists and mental health professionals to provide comprehensive care to individuals with hyperthyroidism and comorbid psychiatric symptoms. Further research is needed to elucidate the precise mechanisms underlying the connection between an overactive thyroid and an overactive mind, ultimately improving the quality of care for affected individuals.
